# Multilayer Graphene/Carbon Black/Chlorine Isobutyl Isoprene Rubber Nanocomposites

**DOI:** 10.3390/polym8030095

**Published:** 2016-03-22

**Authors:** Daniele Frasca, Dietmar Schulze, Volker Wachtendorf, Bernd Krafft, Thomas Rybak, Bernhard Schartel

**Affiliations:** Bundesanstalt für Materialforschung und–prüfung (BAM), Unter den Eichen 87, 12205 Berlin, Germany; daniele.frasca@bam.de (D.F.); dietmar.schulze@bam.de (D.S.); volker.wachtendorf@bam.de (V.W.); bernd.krafft@bam.de (B.K.); thomas.rybak@bam.de (T.R.)

**Keywords:** nanocomposites, rubber, multilayer graphene, carbon black

## Abstract

High loadings of carbon black (CB) are usually used to achieve the properties demanded of rubber compounds. In recent years, distinct nanoparticles have been investigated to replace CB in whole or in part, in order to reduce the necessary filler content or to improve performance. Multilayer graphene (MLG) is a nanoparticle made of just 10 graphene sheets and has recently become commercially available for mass-product nanocomposites. Three phr (part for hundred rubbers) of MLG are added to chlorine isobutyl isoprene rubber (CIIR)/CB composites in order to replace part of the CB. The incorporation of just 3 phr MLG triples the Young’s modulus of CIIR; the same effect is obtained with 20 phr CB. The simultaneous presence of three MLG and CB also delivers remarkable properties, e.g. adding three MLG and 20 phr CB increased the hardness as much as adding 40 phr CB. A comprehensive study is presented, showing the influence on a variety of mechanical properties. The potential of the MLG/CB combination is illustrated to reduce the filler content or to boost performance, respectively. Apart from the remarkable mechanical properties, the CIIR/CB/MLG nanocomposites showed an increase in weathering resistance.

## 1. Introduction

Carbon black (CB) is largely used as filler to improve the performance of rubber composites. CB is produced by the partial combustion or thermal cracking of heavy petroleum products or natural gas. The fine particles of CB always form aggregates and agglomerates [1] and high filler loadings (>30 phr) are usually needed to obtain the mechanical performance desired for elastomer composites [2,3].

Ever since Toyota presented layered silicate/polyamide nanocomposites in the early 1990s [4], polymer research has been concentrating on nanocomposites, and several nanoparticles have been used to reinforce rubbers at even low concentrations. Typical nanofillers include: layered silicates [5,6], spherical nanosilica [7,8], carbon nanotubes [9,10], organically modified clay [11,12,13] and bionanofillers [14,15]. The discovery of graphene [16] has created a new potential nanofiller for polymer nanocomposites [17,18]. Graphene is the 2-D carbon allotrope consisting of a sheet of sp2 carbon atoms arranged in a honeycomb structure [19].

In this study, multilayer graphene (MLG) was used as a nanofiller. It presents a large specific surface area BET (Brunauer Emmett Teller): 250 m^2^/g. This parameter describes quite well the degree of exfoliation and thus the number of layers in graphene stacks [20,21]. A single graphene sheet has a BET of about 2600 m^2^/g. Therefore the MLG used is composed of approximately 10 graphene sheets. Recently, MLG has become commercially available at a reasonable price by applying a modified Hummer method. Materials with a specific surface area BET between 80 and 200 m^2^/g and then stacks consisting of more than 15 sheets are frequently referred to as graphene in the literature. However, we would like to use the following denotation: graphene (less than 7 layers), MLG (7–15 layers) and expanded graphite (15–75 layers) [22,23,24,25]. Even low loadings of MLG already reinforce the final properties of rubber nanocomposites [26,27].

The rubber tested was chlorine isobutyl isoprene rubber (CIIR), the chlorinated form of isobutylene isoprene rubber (IIR), or butyl rubber for short. It is a copolymer of isobutylene (97%–98%) and a small amount of isoprene (2%–3%). CIIR was developed to increase the curing rate of IIR, allowing contemporary vulcanization with natural rubber and styrene-butadiene rubber. Because CIIR presents a very airtight structure, it is the most important rubber for the inner linings of tubeless tires today [28].

The combination of nanoparticles and traditional fillers is a reasonable approach to exploit nanocomposites in usual industrial applications. Thus, in this study, 3 phr of MLG were added to CIIR/CB composites in order to replace CB in part or to boost performance, respectively. The CB used has a specific surface area BET between 70 and 100 m^2^/g. The CIIR/CB compounds were prepared by melt-compounding using a two-roll mill. The compounds with MLG were prepared by pre-mixing MLG with CIIR by an ultrasonically-assisted solution mixing procedure followed by two-roll milling [29].

A low loading of 3 phr MLG was used and its significant influence on the rheological, curing and mechanical properties of CIIR and CIIR/CB composites investigated. Improvements were found, such as the increase in Young’s modulus by a factor of 3 and the replacement of 20 phr CB.

Rubbers are very sensitive to weathering exposure: the combination of oxidative gases and UV degrades the elastomer matrix through multi-step photo-oxidation [30]. The UV absorption and radical scavenging of both CB and MLG was addressed and the improved weathering resistance of the CIIR/CB/MLG nanocomposites discussed.

## 2. Materials and Methods

### 2.1. Materials

CIIR (Chlorobutyl 1240), zinc oxide (Zincoxyd Activ), and mercaptobenzthiazole disulfide (MBTS, Vulcacit DM/C-MG) were obtained from LANXESS Deutschland GmbH, Leverkusen, Germany. Commercially available MLG (EXG R98 250) was produced by Graphit Kropfmühl AG, Hauzenberg, Germany. CB660 (CXN660) and CB (CXN330) were supplied by Orion Engineered Carbons GmbH, Frankfurt, Germany. Stearic acid (stearic acid pure) was produced by Applichem, Darmstadt, Germany. Sulfur was obtained from Merck, Germany. Struktol (Struktol 40 MS Flakes) was supplied by Schill + Seilacher, Böbligen, Germany. Analytical-degree toluene was obtained from Fisher Chemical, Schwerte, Germany.

### 2.2. Preparation of the CIIR Compounds

MLG was dispersed in a toluene/CIIR solution using a sonicator (UPS 400S, Hielscher, Teltow, Germany) for 3 h. Then the mixture was stirred for 2 h. The ratio of elastomer to MLG was 7:1 and the concentration of MLG in the solution was 1 mg/mL. The master batch was obtained after evaporation of the solvent (60 °C, 150 mbar) using a rotary evaporator (Hei Vap Value, Hiedolph, Schwabach, Germany).

CIIR and the other ingredients, as listed in Table 1, were mixed directly in a two-roll mill (Lab Walzwerk MT 6′′ × 13′′, Rubicon, Halle, Germany). The compounds were prepared in three stages. In the first stage, CIIR was mixed with zinc oxide, stearic acid, CB660 and Struktol. In the second stage, the CIIR/MLG master batch or CB was added to the rubber compound. In the third stage, the curatives (sulfur and MBTS) were added. For the compounds without MLG and CB, the second stage was not performed. For all compounds, the rolls were set to a temperature of 50 °C, a speed of 19 RPM a friction ratio of 1.1:1 and a mixing time of 20 min.

The curing time (t100) was obtained by Dynamic Moving Die Rheometer (D-MDR 300, Montech Werkstoffprüfmaschinen, Buchen, Germany). It was 20 min for samples of 2-mm thickness and 25 for 6-mm thickness; the samples were vulcanized at a pressure of 300 bar and a temperature of 180 °C.

### 2.3. Characterization

UV-Vis absorption of freshly produced aqueous dispersions of CB (0.010 and 0.015 mg/mL), MLG (0.005 mg/mL) and their mixture (CB = 0.010 mg/mL plus MLG = 0.005 mg/mL) were measured with a Cary 300 Scan (Varian, Sidney (New South Wales), Australia) double monochromator double channel spectrometer in quartz cuvettes. For the measurement, the cuvettes were placed in front of a LabSphere^®^ DRA-30I integrating sphere, which was used as samples producing stray light were investigated. The wavelength range was 800 to 220 nm with a step width 1 nm. MLG and CB were sonicated in water for 2 h. A baseline correction was carried out using a cuvette filled with pure water.

The radical oxidation of cumene (10 mL) was performed to determine the radical scavenging behavior of the tested carbon particles; AIBN (10 mg) was the initiator [31]. The studied reaction consists of 3 phases:

Initiation: AIBN ➔ r^•^ + RH ➔ R^•^
Propagation: R^•^ + O_2_ ➔ RO_2_^•^ + RH ➔ ROOH + R^•^
Termination: 2 RO_2_^•^ ➔ inactive products
(RH = cumene, R^•^ = cumylalkyl radical, RO2^•^ = cumylperxoy radical, ROOH = cumylhydroperoxide).

At 60 °C, the initiator (AIBN) decomposed into radicals (initiation). Then, the radicals reacted with cumene. This reaction resulted in cumene alkyl radicals, which were oxidized by oxygen into cumylperxoy radicals in the propagation stage. Furthermore, cumylperxoy radicals reacted with cumene, forming other cumene alkyl radicals. When cumylperoxy radicals reacted with each other, the reaction ended (termination).

MLG (5 mg, 30 mg) and CB (30 mg) were sonicated 10 minutes in the cumene. Than AIBN was added to the MLG/cumene dispersion and the oxygen consumption was controlled by measuring the pressure decrease in the closed air volume above the reaction mixture.

Using a Dynamic Moving Die Rheometer (D-MDR 3000, Montech Werkstoffprüfmaschinen), the dynamic viscosity (η’) of the uncured samples (5 g) was measured as a function of frequency. The temperature was 100 °C and the strain amplitude was 1%. The storage modulus (G’) as a function of the amplitude was also measured with a Dynamic Moving Die Rheometer (D-MDR 3000, Montech Werkstoffprüfmaschinen) on the uncured samples (5 g). The temperature was 60 °C and the frequency 1 Hz.

Scanning electron microscopy (SEM) micrographs of the freeze-fractured gold-coated surfaces of vulcanized samples were taken with a scanning electron microscope (Zeiss EVO MA 10) using an acceleration voltage of 10 kV. The micrographs of CB and MLG were taken without gold sputtering.

The samples (80 nm thick), for the TEM micrographs were prepared using a cryo microtome (Ultracut UCT, Leica, Wetzlar, Germany) at −100 °C. The TEM micrographs of CIIR-MLG-3 were taken with JEM-2200 FS (Jeol, Peabody, MA, USA); the acceleration voltage was 200 kV.

Tensile tests were performed on 5 dumbbell specimens (2-mm thickness) according to DIN 53504; Young’s modulus tests were performed on 3 dumbbell specimens (2-mm thickness) according to ISO 527; and shore A hardness measurements were performed according to ISO 7619-1 on 3 samples of 6-mm thickness.

The storage modulus (G′) and dynamic loss factor (tan δ) were measured on 2 samples of 2-mm thickness as a function of temperature using an MCR 501 Rheometer (Anton Paar, Ostfildern, Germany). The frequency was 1 Hz, the strain amplitude was 0.1%, the temperature range of −80 to 70 °C and the heating rate was 1 °C/min.

The weathering/ageing process of dumbbell 10 test specimens (2 mm thickness) was conducted using the 24 h weathering cycle in Table 2 repeatedly conducted over 1000 h (for one half of the samples) and 1500 h (for the other half), respectively. The conditions of the cycle contain a step at −10 °C, which could bring mechanical tension into the sample, as well as rain phases, which can cause extraction of soluble or dispersible components off the system. Weathering was carried out using a fluorescent UV lamp device of the type Global UV Test 200 (Weiss Umwelttechnik GmbH, Reiskirchen, Germany), according to ISO 4892-3. The spectral distribution—characterized by UVA-340 nm fluorescent lamps (ISO 4892-3, type 1A) and spectrally neutral filtering using a PVDF-membrane in the device’s door—was measured in the sample plane by means of a MSS 2040 spectro-radiometer. As the spectral distribution from the fluorescent lamps is limited to UV and near VIS, radiation heating can be neglected (TSurface − TChamber < 2 K). Thus, the degradation-relevant temperature can be controlled very closely over a wide range. The device allows full humidity control and uses water spraying for the wetting phases. UV-irradiance was 40 W/m^2^.

## 3. Results

### 3.1. Characterization of MLG and CB

**Figure 1:**
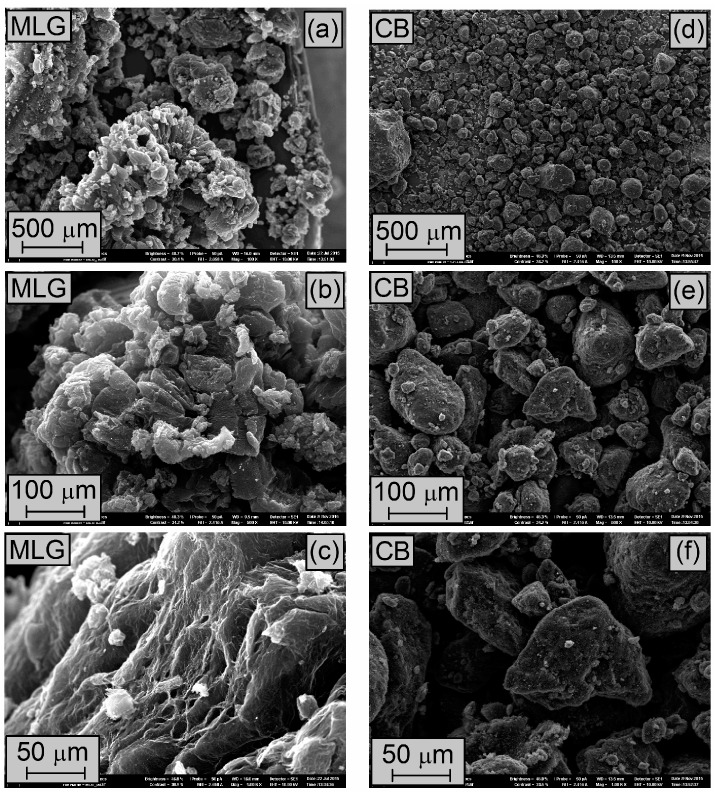
SEM micrographs of (**a**–**c**) MLG and (**d**–**f**) CB.

The left side of Figure 1 shows the SEM micrographs of the MLG used. The MLG particles had different shapes, but most of them present a worm-like shape (Figure 1a). Some particles are small with a diameter about 50 μm and others very big with a length of about 1 mm. Nevertheless, each particle consists of several MLG stacks (Figure 1b). Figure 1c shows the highly delaminated structure of MLG, in good correspondence to the high BET.

The SEM micrographs of CB are shown in the right side of Figure 1. The CB particles presented a spherical shape with diameters between 20 and 100 μm. Furthermore, the surface of the particles turns out to be completely smooth. At this magnification, no aggregates of CB particles were observed.

**Figure 2:**
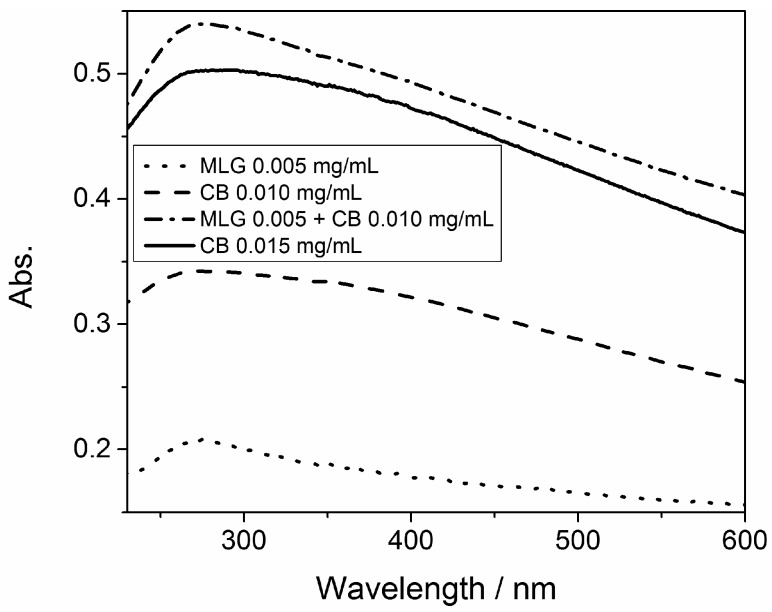
UV-Vis absorption of water dispersions of CB, MLG and their mixture.

Figure 2 reports the UV-Vis absorption of aqueous dispersions of CB, MLG and a mixture of the two carbon particles. All of the spectra presented a maximum of absorption of about 270 nm, which corresponds to the electronic transition from the bonding orbital π to the anti-bonding orbital π* [32]. This transition is typical for carbon particles like MLG and CB [33]. The UV band of the π-π* transition is more defined in the spectrum of MLG (0.005 mg/mL), where it is almost a peak, than in the spectra of CB (0.010 and 0.015 mg/mL), where it is large and rough . MLG consists of only sp2 carbon atoms, whereas CB is made of sp2 and sp3 carbon atoms; in the presence of sp3 carbon atoms the UV band caused by π-π* transition is reduced [34]. The spectra of the CB/MLG mixture presented an evident peak at about 270 nm because of the presence of the tested nanoparticle. Moreover, the absorption of the mixture, with a final concentration of 0.015 mg/mL, was higher than for the same concentration of CB. Hence, MLG presented a higher UV-Vis. absorption than CB.

**Figure 3:**
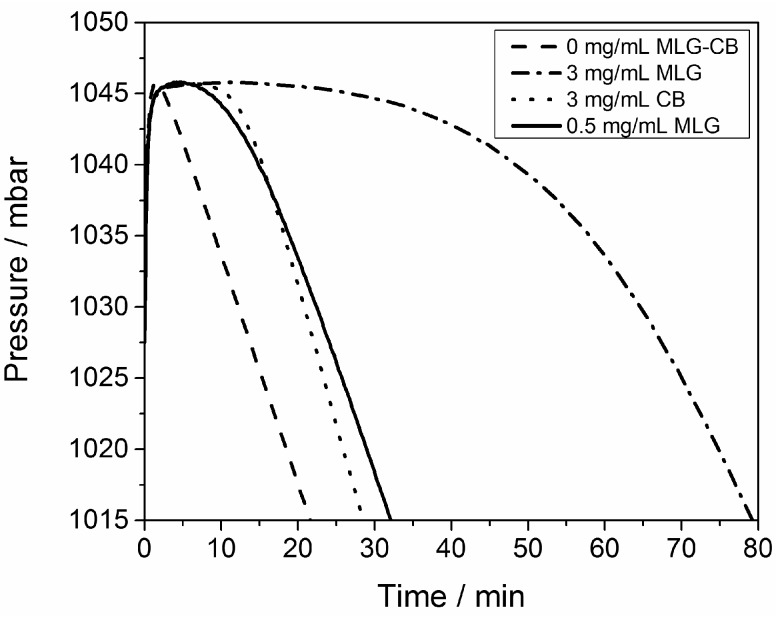
Change in pressure during the cumene oxidation with and without MLG or CB.

The radical scavenging efficiency of the CB and MLG was determined by studying the radical oxidation of cumene [35], which is thought to be a model for autoxidative radical oxidation of the elastomer. When this reaction occurs, it consumes oxygen, which results in a reduction of the pressure, as shown in Figure 3. The reaction started in a few minutes without MLG and CB. In the presence of CB and MLG, the reaction started after an induction time: 11 min for MLG and 13 min for CB, with the induction time as a parameter of the stabilization present in the system. The concentration of MLG was 0.5 mg/mL, while the concentration of CB was six times higher (3 mg/mL). The two carbon particles inhibited the oxidation of cumene because they intercepted the cumene alkyl radicals. Moreover, after the induction time, the pressure reduced more slowly in the presence of MLG than with CB. The radical scavenging efficiency of MLG increased with the concentration of the nanoparticle. The induction time with a concentration of 3 mg/mL of MLG was about 50 min.

MLG was more efficient than CB because of its larger surface area and its chemical structure made only of sp2 carbon atoms. In this hybridization the carbon atoms have a free π orbital. The electrons of the radicals were delocalized in the free π orbital of MLG. The carbon atoms of carbon black present sp2 and sp3 hybridization, and thus fewer free π orbitals than MLG.

### 3.2. Rheological Properties of the Uncured Systems

**Figure 4:**
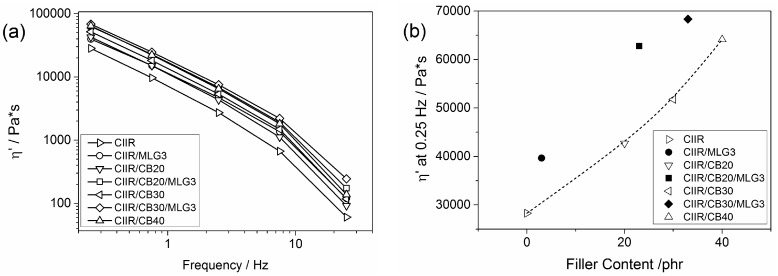
(**a**) Dynamic viscosity as a function of the frequency of CIIR and its composites; and (**b**) dynamic viscosity at 0.25 Hz as a function of the filler content; the line is a visual guide.

Figure 4a shows the dynamic viscosity (η′) of the uncured systems as a function of the frequency. The frequency sweep shows a decrease in η′ for higher frequencies, because the elastic behaviour loses importance. CB and MLG reinforced CIIR in terms of η, as reported by Kumar *et al.* [36]; in fact, the curves of the filled systems show a remarkable shift to higher values in the logarithmic presentation compared to CIIR.

In Figure 4b, the η’ at 0.25 Hz is plotted as a function of the filler content. The reinforcement of 3 phr MLG in η‘ corresponded to the reinforcement of more than 10 kPas and thus to an equivalent of 18 phr CB. The viscosity η’ increased with CB content: 20, 30 and 40 phr of CB resulted in reinforcing effects of 34%, 45% and 56%, respectively. The reinforcing effect of CB was increased by MLG. The combination of 20 phr CB and 3 phr MLG increased η’ by around 20 kPas and thus like 39 phr CB; hence, in this case, 3 phr MLG replaced 19 phr CB. The value of η’ at 0.25 Hz of CIIR/CB30/MLG3 was the highest and equal to that for 46 phr CB. As to the effect on η’, 3 phr MLG replaced at least 15 phr CB in CIIR/MLG/CB nanocomposites. The efficiency of the nanofiller was somewhat higher in combination with a low CB loading.

**Figure 5:**
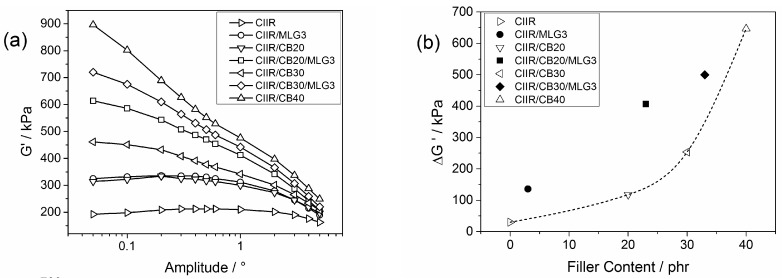
(**a**) Storage modulus as a function of the strain amplitude of CIIR and its composites; and (**b**) difference between initial and final storage modulus as a function of the filler content; the line is a visual guide.

Figure 5a shows the G’ of the uncured systems as a function of the strain amplitude. Usually, the G’ values of unfilled rubber systems do not change along with amplitude. For the filled system, G’ increases with the decreasing amplitude because of the formation of a filler network [37]. This behaviour of filled rubber systems is known as the “Payne effect”. In fact, no “Payne effect” was observed for CIIR and it was small for CIIR/MLG3, CIIR/CB20 and CIIR/CB30. The “Payne effect” becomes evident at higher filler loadings, as reported by Fritzsche *et al.* [38], and hence for CIIR/CB20/MLG3, CIIR/CB30/MLG3 and CIIR/CB40.

Figure 5b reports the difference between the maximum and minimum of G′ as a function of the filler content. ΔG′ of CIIR/MLG3 was 136 kPa, which is in correspondence with 23 phr CB. Adding 3 phr MLG/20 phr CB shows the largest increase in ΔG (around 300 kPa) and thus reinforced ΔG′ as much as adding 32 phr CB; consequently the effect of 3 phr MLG was similar to 12 phr CB. In the presence of 30 phr CB, the effect of 3 phr MLG corresponded to 7 phr CB. Due to the non-linear behaviour of ΔG′ *versus* CB content, the efficiency of adding 3 phr MLG with respect to replacing CB decreased markedly with higher CB content.

### 3.3. Curing Properties

**Figure 6:**
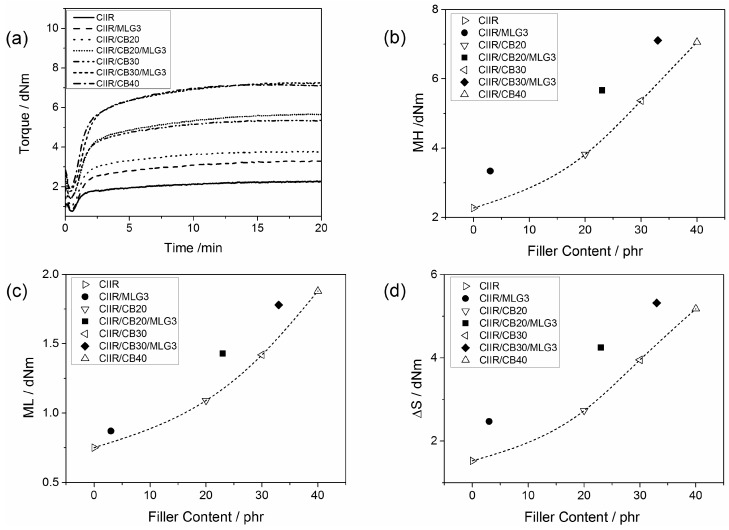
(**a**) Curing curves of CIIR (chlorine isobutyl isoprene rubber) and its composites; (**b**) maximum of the torque; (**c**) minimum of the torque; and (**d**) difference between maximum and minimum of the torque as a function of the filler content; the lines are visual guides.

The curing curves of CIIR and its composites with MLG and CB are reported in Figure 6a. Vulcanization results in an increase of the torque over time. MLG and CB reinforced the torque and thus the curves of the filled compound were higher than CIIR.

Figure 6b reports the maximum of torque (MH) as a function of the filler content. MH is a measure of the stock modulus of the cured compounds [39]. Adding 3 phr MLG increases the MH of the composites by 1 to 2 dNm. Adding 3 phr MLG increased the MH of CIIR as much as 16 phr CB. In the presence of 20 and 30 phr CB, the reinforcing effect of 3 phr MLG was similar to 12 phr CB.

Figure 6c shows the minimum of the torque (ML) as a function of the filler loading. ML is a measure of the viscosity of the uncured compounds [40]. Adding 3 phr MLG increased the ML of CIIR by 14 %, which matched the effect of 10 phr CB. A similar effect was recorded in the presence of 20 phr CB. In the case of CIIR/CB30/MLG3, the nanofiller reinforced like 8 phr CB.

The difference between MH and ML (Δ*S*) is usually assumed to be proportional to the cross-link density [41,42]. Δ*S* is plotted as function of the filler content in Figure 6d. Adding 3 phr MLG increased ΔS by 1 to 1.4 dNm. As for MH, the effect of 3 phr MLG in CIIR/MLG was similar to 16 phr CB. The presence of 20 and 30 phr CB in CIIR/MLG/CB nanocomposite showed the same effect as adding an additional 12 phr CB. The curing properties of CIIR and its composites are summarized in Table 3.

### 3.4. Morphology of the CIIR and its Composites

**Figure 7:**
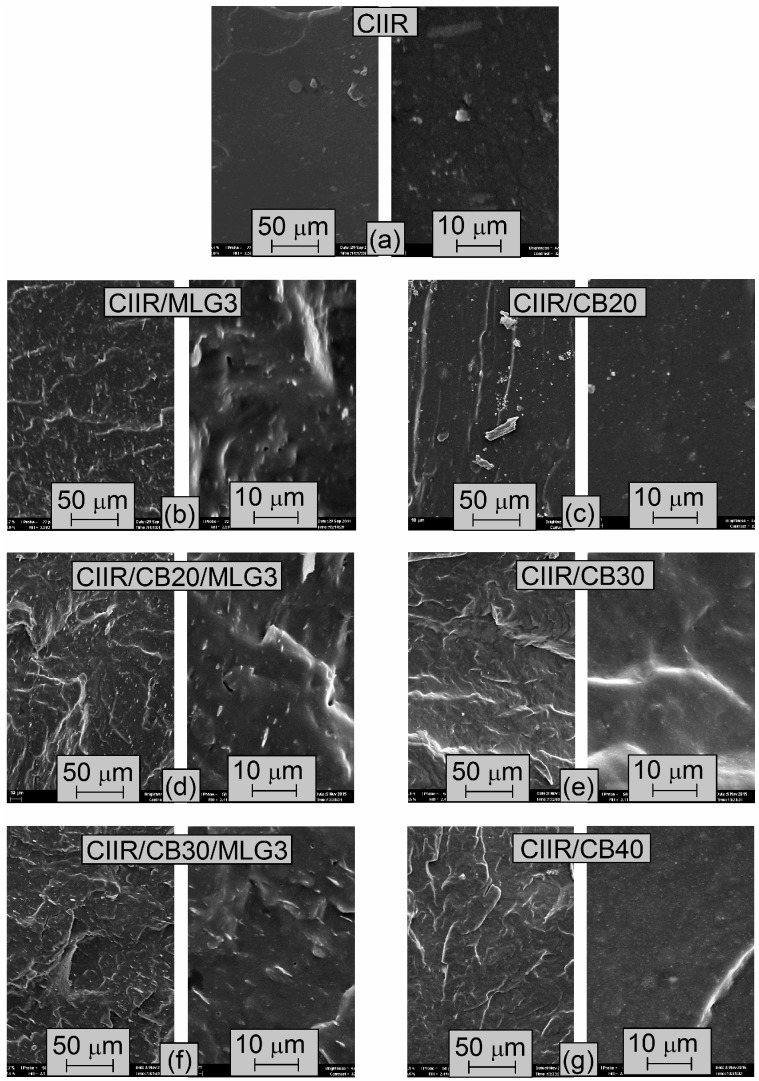
(**a**)–(**g**) SEM micrographs of CIIR and its composites.

Figure 7 shows the SEM micrographs of the freeze-fractured surface of CIIR and its composites. The surface of CIIR was smooth (Figure 7a). The incorporation of 3 phr MLG resulted in an increase in roughness of the surface of the investigated rubber (Figure 7b). In contrast, the surface of CIIR/CB20 presented few and large protuberances, but was completely smooth at high magnifications (Figure 7c). CIIR/CB20/MLG3 and CIIR/CB30/MLG/3 had a rough surface (Figure 7f). The small protuberances, visible at higher magnifications, were attributed to the MLG wrapped by a layer of rubber [43,44]. In fact, they are also presented on the surface of CIIR/MLG3. The surfaces of CIIR/CB30 and CIIR/CB40 were also rough (Figure 7e,g). As for CIIR/CB20, they were almost smooth at higher magnifications. Agglomerates were not detected on the fractured surfaces of the CIIR composites because the fillers were well dispersed in the elastomeric matrix.

**Figure 8:**
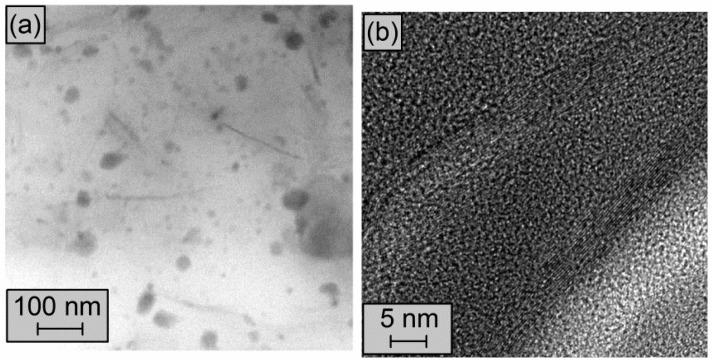
(**a**) and (**b**) TEM micrographs of CIIR/MLG3.

During the preparation of the nanocomposites the particles of MLG (Figure 1a–c) were broken apart completely. Then the single MLG stacks were homogenously dispersed in the elastomeric matrix as shows the TEM micrograph of CIIR/MLG3 (Figure 8a), where the black lines were identified as MLG while the black spots were allocated to CB and zinc oxide. Moreover any prevalent orientation was detected.

The TEM micrograph in Figure 8b shows two MLG stacks, which consist of 12 graphene sheets. Examining the TEM micrographs the approximate dimensions of the dispersed MLG were determined: 5 ± 2 nm of thickness and 170 ± 60 nm of width. Therefore the average aspect ratio of MLG in CIIR nanocomposite was 34.

### 3.5. Mechanical Properties

**Figure 9:**
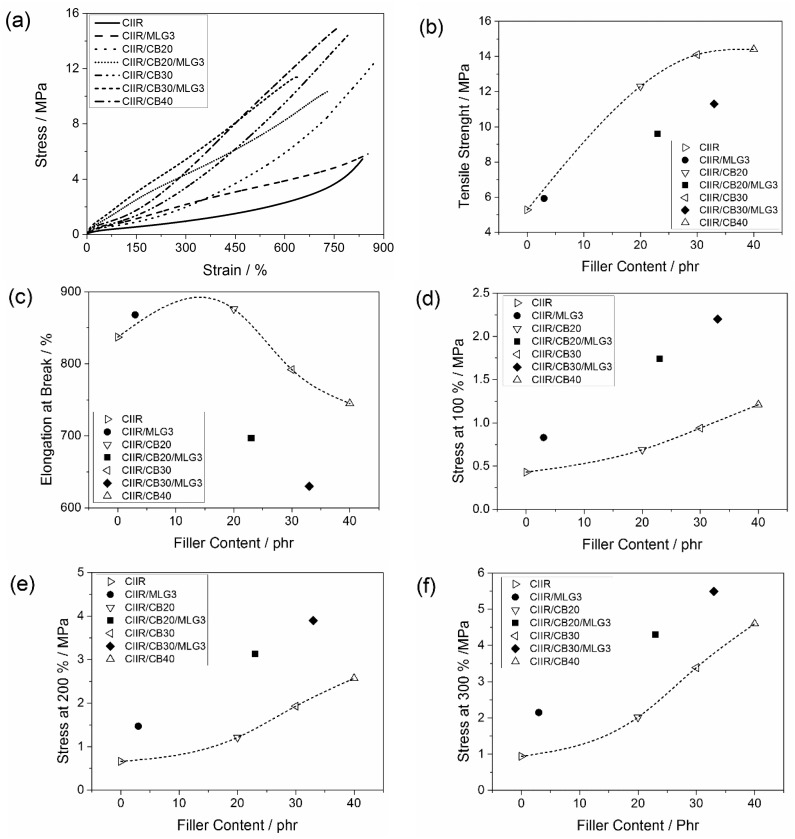
(**a**) Tensile stress *vs.* strain curves and of CIIR and its composites; (**b**) tensile strength; (**c**) elongation at break; (**d**) stress at 100% of elongation; (**e**) stress at 200% of elongation; and (**f**) stress at 300% of elongation as functions of the filler content. The lines are visual guides.

The stress-strain curves of CIIR and its composites are reported in Figure 9a. The mechanical properties of the elastomer were reinforced by CB and MLG; thus the composites were stiffer than CIIR. The shape of the curves for CIIR/CB20, CIIR/CB30 and CIIR/CB40 is almost the same: a gentle increase in the stress at low elongation and a strong one at high elongation. On the other hand, the curves of CIIR/CB20/MLG3 and CIIR/CB30/MLG3 are almost linear. CIIR/CB20/MLG3 and CIIR/CB30/MLG3 were the stiffest composites. Moreover, up to 400% elongation the stresses of CIIR/MLG3 and CIIR/CB20 were very similar and CIIR/CB30/MLG3 presented the highest stress up to 450% elongation.

In Figure 9c–f, the mechanical properties were plotted as function of filler loading. The final tensile strength of CIIR was not significantly increased by 3 phr MLG, while it was strongly increased by 20 and 30 phr CB (Figure 9b). The effect of 30 phr CB was very similar to 40 phr CB. The combinations of CB and MLG also resulted in a higher tensile strength of CIIR, but this increase was lower than the effect of 20 phr CB. CIIR/CB20/MLG3 presented a tensile strength like a compound with 10 phr CB, while the combination of 30 phr CB and 3 phr MLG reinforced the tensile strength as much as about 15 phr CB.

Figure 9c shows the elongation at break as a function of the filler content. Adding 3 phr MLG and 20 phr CB resulted in a small increase in the final elongation, while the compounds with higher filler loading were more brittle. The reduction in elongation at break due to 3 phr MLG plus 20 phr CB was similar to that in composites with more than 50 phr CB. The strongest reduction was obtained with the simultaneous presence of 3 phr MLG and 30 CB. Based on a rough extrapolation, it was similar to more than 65 phr CB.

The effect of the 3 phr on the stress at 100 % was very strong, as shown in Figure 9d. The stress at 100% of CIIR/MLG3 was the same as for a CIIR compound with 25 phr CB. The reinforcement of 3 phr MLG was even stronger in the presence of CB. The stress was increased by up to more than a factor 2 when comparing CIIR/MLG/30 phr CB with CIIR/30 phr CB. Using a rough extrapolation, the combination of 3 phr MLG with 20 phr CB and 30 phr reinforced to the same degree as more than 55 phr CB and more than 65 phr, respectively.

A similarly strong increase in stress by up to more than a factor of 2 was observed for 200% elongation, when CIIR/MLG/ CB was compared with CIIR/ CB for 20 phr CB and 30 phr CB. The stress at 200% of CIIR/MLG3 was similar to a composite with 25 phr CB (Figure 9e). The simultaneous reinforcement of 3 phr MLG and 20 phr was probably similar to more than 45 phr CB. The stress at 200% of CIIR/CB30/MLG3 was roughly the same of a composite with more than 55 phr CB.

Figure 9f shows the stresses at 300% as a function of the filler content. Adding 3 phr MLG increases the stress by a factor of *ca*. 2. The stress at 300% of CIIR/MLG3 was the same as for compounds with 21 phr CB. In combination of 20 and 30 phr CB, 3 phr MLG improved the stress at 300% like 17 phr CB.

**Figure 10:**
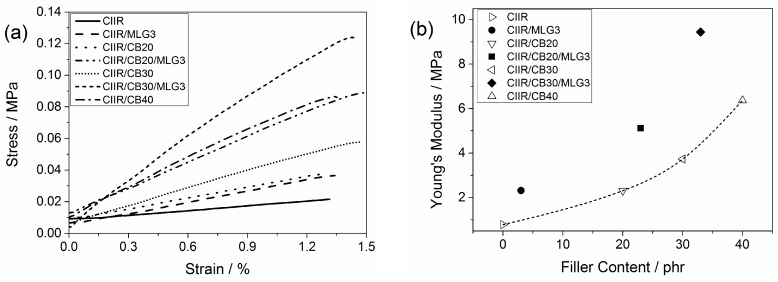
(**a**) Stress *vs.* strain curves to determine Young’s modulus of CIIR and its composites; and (**b**) Young’s modulus as a function of the filler content. The line is a visual guide.

Figure 10 shows the stress-strain curves to determine the Young’s modulus of CIIR and its composites. The Young’s modulus is the slope of the curves and it was increased by adding CB and MLG. In Figure 10b the elastic moduli are plotted as a function of the filler content. Adding 3 phr MLG tripled the Young’s modulus of CIIR. Thus 3 phr MLG reinforced CIIR as much as adding 20 phr CB (Figure 9b). An amount of 3 phr MLG in CIIR/CB composites reinforced the Young’s modulus by 2.8 MPa and 5.7 MPa for CIIR/20 phr CB and CIIR/30 phr CB, respectively. They match the effect of adding 17 phr CB in addition to the 20 and 30 phr CB.

**Figure 11:**
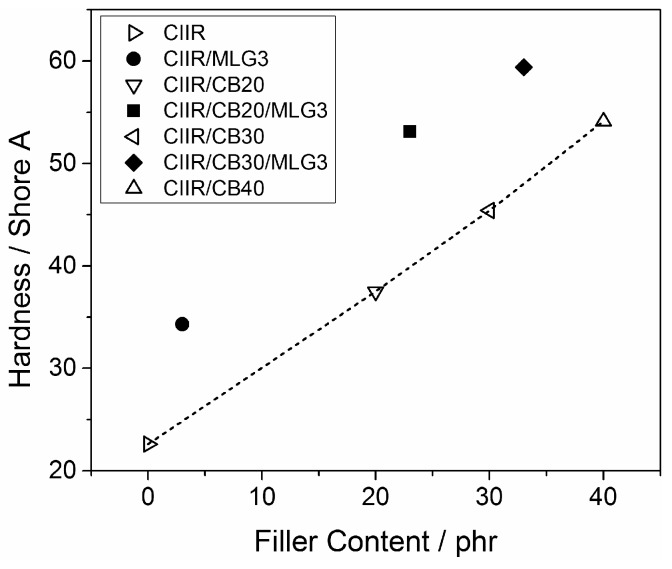
Hardness of CIIR and its composites as a function of the filler content; the line is a visual guide.

Figure 11 reports the hardness of CIIR and its composites as a function of filler content. The hardness of CIIR of 12 Shore A was increased by just 3 phr MLG. This effect was similar to that of 17 phr CB. MLG also strongly reinforced the rubber in the presence of CB. In fact, the hardness of CIIR/CB20/MLG3 (53 Shore A) was very similar to the hardness of CIIR/CB40 (54 Shore A). The increase in Shore A hardness was linear with CB content and quite constant (13.8 ± 1.1) when adding 3 phr MLG. Hence, in the case of hardness, 3 phr MLG replaced about 20 phr CB in all of the nanocomposites. CIIR/CB30/MLG3 was the hardest compound. The mechanical properties of CIIR and its composites are summarized in Table 4.

### 3.6. Dynamic Mechanical Properties

**Figure 12:**
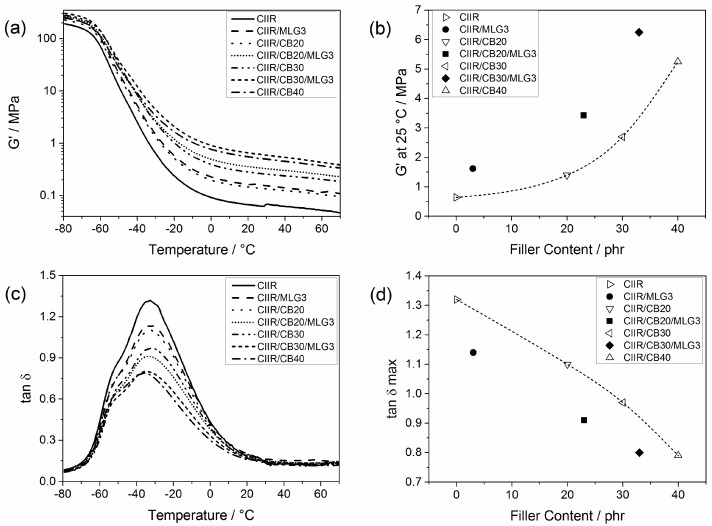
(**a**) Storage modulus of CIIR and its composites as a function of the temperature; (**b**) storage modulus at 25 °C as a function of the filler content—the line is a visual guide; (**c**) tan δ of CIIR and its composites as a function of temperature; and (**d**) maximum of tan η as a function of the filler content—the line is a visual guide.

The dynamic mechanical properties of CIIR and its compounds are shown in Figure 12. The storage modulus (G′) as a function of the temperature is reported in Figure 12a. The reinforcing effect of the tested fillers resulted in an increase of G′ in the solid state at low temperatures as well as in the rubber state at temperatures above −30 °C. In all temperature ranges, the G′ of CIIR/MLG3 and CIIR/CB20 are almost identical. Higher filler content results in a higher increase in G′. Figure 12b reports G′ at 25 °C as a function of the filler content. At this temperature, the G′ of CIIR/MLG3 was increased by more than a factor of 2 compared to the G′ of CIIR, and thus presented the same G′ as a CIIR compound with 23 phr CB. In combination with 20 and 30 phr CB, 3 phr MLG also increased the G´ by more than a factor of 2, which corresponds to the increase in G’ at 25 °C for adding another *ca*. 13 phr CB.

Figure 12c reports the loss factor peak (tan δ of CIIR and its composites. The presence of fillers resulted in a reduction in the height of the peak because the fillers decreased the elastomeric chain mobility [45]. Strong rubber–filler interactions are the reasons for this behavior [46,47].

The maximum of tan δ as a function of the filler loading is reported in Figure 11d. The effect of 3 phr MLG was the same as of 16 phr CB. Adding 3 phr MLG reduced the maximum of tan δ of CIIR like 13 phr CB in the presence of 20 phr CB, while in the case of CIIR/CB30/MLG3, 3 phr MLG acted like 10 phr MLG.

### 3.7. Durability of Mechanical Properties Against Weathering Exposure

**Figure 13:**
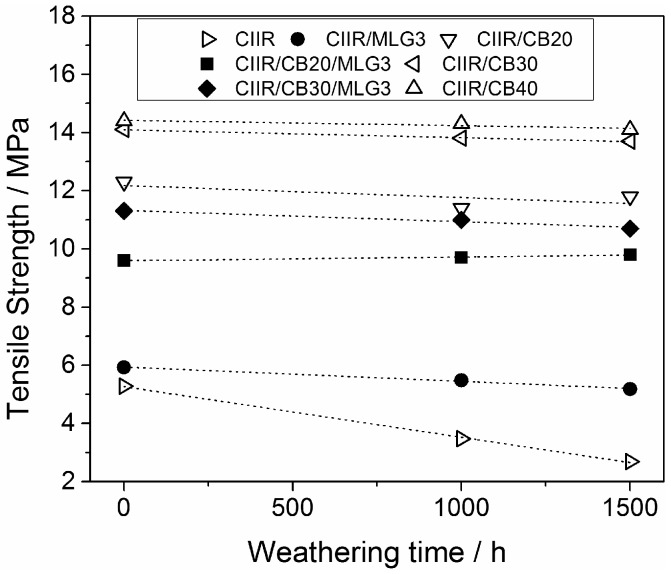
Tensile strength of CIIR and its composites as a function of the weathering/exposure duration time. The lines are visual guides.

Figure 13 shows the tensile strength of CIIR and its composites with MLG and CB as a function of the weathering. In the case of unfilled CIIR the tensile strength was reduced by 34% after 1000 h and by 49% after 1500 h. The combination of oxidative gases and UV degrades the CIIR through multi-step photo-oxidation [30]. This degradation is the cause of the loss in tensile strength. The CIIR composites reinforced with MLG and CB conserved their initial tensile strength after the weathering/ageing because the two fillers inhibited the photo-oxidation. As described in Section 3.1, MLG and CB absorb UV and act as radical scavengers. These two mechanisms explain the stabilization effect of the carbon particles studied.

## 4. Conclusions

MLG is a nanoparticle consisting of just approximately 10 graphene sheets and it was proposed as a nanofiller for CIIR in order to partly replace CB. The incorporation of MLG significantly improved the curing, rheological and mechanical properties of CIIR, e.g., just 3 phr CB increased the Young’s modulus of CIIR by a factor of 3. Thus, the effect is similar to adding 20 phr CB. The strong reinforcing effect of MLG was also evident in the presence of CB, as shown for a variety of mechanical characteristics in this comprehensive investigation. For instance, the combination of 3 phr MLG and 20 phr CB in CIIR/MLG/20 phr CB increased the Shore A hardness of CIIR by 14 compared to CIIR/20 phr CB, achieving the same hardness as CIIR/40 phr CB. A similarly large impact of adding 3 phr MLG on properties of CIIR/CB composites was observed for all of the properties investigated, which were often improved by a factor of 2 to 3. The influence of 3 phr MLG mostly equals a CB amount of 10–25 phr, sometimes even more. The tested carbon particles absorbed UV, acted as radical scavengers and hence inhibited the weathering degradation of CIIR. Therefore the CIIR composites with MLG and CB conserved their initial mechanical properties after weathering exposure.

This study proposes the combination of CB composites with MLG nanocomposites as promising route to replace some of the CB as a filler. Rubber/CB/MLG compounds harbor the potential for reducing the filler amount and outperform the rubber/CB composites. It should not escape our notice that the investigation of this potential is not only an academic indulgence, but this is also ready for industrial and commercial exploitation.

## Figures and Tables

**Table 1:** Formulation of the CIIR (chlorine isobutyl isoprene rubber) compounds in parts per hundred of rubber (phr).

Ingredients	CIIR	CIIR/MLG3	CIIR/CB20	CIIR/CB20/MLG3	CIIR/CB30	CIIR/CB30/MLG3	CIIR/CB40
CIIR	100	100	100	100	100	100	100
Zinc oxide	3.0	3.0	3.0	3.0	3.0	3.0	3.0
Stearic acid	2.0	2.0	2.0	2.0	2.0	2.0	2.0
CB660	0.5	0.5	0.5	0.5	0.5	0.5	0.5
Struktol	7.0	7.0	7.0	7.0	7.0	7.0	7.0
Sulfur	0.5	0.5	0.5	0.5	0.5	0.5	0.5
MBTS	1.5	1.5	1.5	1.5	1.5	1.5	1.5
CB	-	-	20	20	30	30	40
MLG	-	3	-	3	-	3	-

**Table 2:** Weathering exposure cycle. Continuous UV irradiation at 40 W/m^2^.

Time/h	Temperature/°C	Humidity	
4	25		Rain
4	80	<10%	
4	25		Rain
4	80	<10%	
4	25		Rain
4	−10	<10%	

**Table 3:** Curing properties of CIIR and its composites.

	ML (dNm)	MH (dNm)	ΔS (dNm)
CIIR	0.75 ± 0.01	2.27 ± 0.01	1.52 ± 0.02
CIIR/MLG3	0.87 ± 0.03	3.34 ± 0.02	2.47 ± 0.05
CIIR/CB20	1.09 ± 0.06	3.82 ± 0.06	2.73 ± 0.01
CIIR/CB20/MLG3	1.43 ± 0.01	5.67 ± 0.01	4.25 ± 0.01
CIIR/CB30	1.42 ± 0.01	5.37 ± 0.01	3.95 ± 0.02
CIIR/CB30/MLG3	1.79 ± 0.10	7.11 ± 0.21	5.32 ± 0.11
CIIR/CB40	1.88 ± 0.04	7.06 ± 0.16	5.18 ± 0.20

**Table 4:** Mechanical properties of CIIR and its composites.

	**Stress 100%/MPa**	**Stress 200%/MPa**	**Stress 300%/MPa**	**Tensile strength/MPa**
CIIR	0.43 ± 0.01	0.66 ± 0.03	0.94 ± 0.04	5.28 ± 0.89
CIIR/MLG3	0.83 ± 0.01	1.47 ± 0.02	2.15 ± 0.03	5.93 ± 0.29
CIIR/CB20	0.69 ± 0.01	1.21 ± 0.02	2.02 ± 0.04	12.30 ± 0.51
CIIR/CB20/MLG3	1.74 ± 0.03	3.13 ± 0.05	4.30 ± 0.05	9.62 ± 0.81
CIIR/CB30	0.94 ± 0.02	1.93 ± 0.04	3.39 ± 0.08	14.10 ± 0.68
CIIR/CB30/MLG3	2.20 ± 0.06	3.90 ± 0.08	5.49 ± 0.96	11.30 ± 0.74
CIIR/CB40	1.21 ± 0.01	2.57 ± 0.04	4.60 ± 0.06	14.40 ± 0.28
	**Elongation at break/%**	**Young’s modulus/MPa**	**Hardness/Shore A**	
CIIR	837 ± 29	0.79 ± 0.22	22.6 ± 0.6	
CIIR/MLG3	868 ± 26	2.32 ± 0.08	34.3 ± 0.9	
CIIR/CB20	876 ± 20	2.31 ± 0.11	37.5 ± 0.3	
CIIR/CB20/MLG3	697 ± 46	5.12 ± 0.18	53.1 ± 0.4	
CIIR/CB30	792 ± 25	3.73 ± 0.05	45.4 ± 0.4	
CIIR/CB30/MLG3	630 ± 38	9.44 ± 0.43	59.4 ± 0.5	
CIIR/CB40	745 ± 16	6.37 ± 0.25	54.1 ± 0.6	
